# Supplementary material for: Antiretroviral therapy initiation and outcomes of hospitalized HIV-infected patients in Uganda—An evaluation of the HIV test and treat strategy
Source: PLoS One. 2022 Aug 19;17(8):e0268122. doi: 10.1371/journal.pone.0268122 (PMC9390910; doi:10.1371/journal.pone.0268122)
Supplement: S2 File — (DOCX) [file pone.0268122.s002.docx]

**APPENDIX 3.1: HEALTH CARE WORKER INTERVIEW GUIDE**

**Title of the proposed study**

**ANTI-RETROVIRAL THERAPY INITIATION AND 30-DAY OUTCOMES AMONG HOSPITALITZED PATIENTS NEWLY DIAGNOSED WITH HIV IN THE TEST AND TREAT ERA**

**Background information**

1. Age
2. Sex
3. Place of work/ institution
4. Occupation
5. Field of specialization
6. Highest level of education attained
7. Duration of service and experience

**Guiding questions**

***1.*** Please share with me what you understand by HIV test-and-treat. ***Probe: pre and post- test counselling, what is done when a person is found positive, patient referral, initial HIV test-and-treat clinic visit and linkage to care/follow up.***

***2.*** When HIV test-and-treat was initiated, were the health workers trained? ***Probe for the kind of training that they received, who trained them and the cadre that was trained. Are there fresher courses,? Is there need for frequent training?***

1. ***As a health practitioner, when do you think a newly diagnosed hospitalised HIV positive individual should be started on ART? WHO stage, CD4, opportunistic infections (TB, CCM, Hepatitis, Kaposi sarcoma) why? Probe more on any reason why not to initiate ART immediately.***
2. **In your opinion, what factors are associated with failure to initiate ART among newly diagnosed hospitalised HIV positive individuals. *Probe for Disclosure of HIV status to partner / relatives, social support, stigma, thinking people do blame HIV infected individuals, patient readiness to start on ART immediately when offered. Inadequate pre and post-test counselling. Other comorbidities other than TB/CCM e.g. feeding issues, Liver, renal, cardiac and dysfunction,***
3. ***What factors facilitate early initiation to ART by newly diagnosed HIV infected people? Probe for Human resource, HIV testing kit availability, Linkage to care post discharge, ART drugs availability, Donor restrictions. How is the ART initiation process is it the doctors decision or the ART nurse/champion to decide who to start***
4. ***Share with me the challenges faced by health workers in delivering HIV test-and-treat among newly diagnosed hospitalised HIV positive individuals. Probe for system factors (Delayed laboratory work up, human resources, donor restrictions,), policy procedures (being a referral hospital, distance, linkage system,), ART stock out, work overload, easiness for the patient to get the ART after doctors’ decision etc.***
5. *What can be done to solve such challenges?*

Thank you Version 1
