# Supplementary material for: Antiretroviral therapy initiation and outcomes of hospitalized HIV-infected patients in Uganda—An evaluation of the HIV test and treat strategy
Source: PLoS One. 2022 Aug 19;17(8):e0268122. doi: 10.1371/journal.pone.0268122 (PMC9390910; doi:10.1371/journal.pone.0268122)
Supplement: Attachment — Submitted filename: Response to Reviewers_PLOS_12_1_22.docx [file pone.0268122.s003.docx]

**Response to Reviewers**

**Comment:** Please review your reference list to ensure that it is complete and correct. If you have cited papers that have been retracted, please include the rationale for doing so in the manuscript text, or remove these references and replace them with relevant current references. Any changes to the reference list should be mentioned in the rebuttal letter that accompanies your revised manuscript. If you need to cite a retracted article, indicate the article’s retracted status in the References list and also include a citation and full reference for the retraction notice.

**Response: Thank you for your comment; I have added one reference,**

Kerschberger, B., Jobanputra, K., & Schomaker, M. (2019). Feasibility of antiretroviral therapy initiation under the treat-all policy under routine conditions: a prospective cohort study from Eswatini. *22*(10), e25401. doi: 10.1002/jia2.25401

**Comment:** We note that the grant information you provided in the ‘Funding Information’ and ‘Financial Disclosure’ sections do not match.

When you resubmit, please ensure that you provide the correct grant numbers for the awards you received for your study in the ‘Funding Information’ section.

**Response: Thank you for your guidance**

**I agree with the following statement financial statement; “The funder provided support in the form of salaries for authors [FCS, AK], but did not have any additional role in the study design, data collection and analysis, decision to publish, or preparation of the manuscript. The specific roles of these authors are articulated in the ‘author contributions’ section. This statement has been adapted in the funding source section page 18.**

**Comment:** Please include captions for your Supporting Information files at the end of your manuscript, and update any in-text citations to match accordingly. Please see our Supporting Information guidelines for more information:

**Response:** Captions of the supporting information files have been put.

**S1 Table.**

**S2 STaTa data set**

**Reviewers’ comments**

**Comment:** Have the authors made all data underlying the findings in their manuscript fully available?  The [PLOS Data policy](http://www.plosone.org/static/policies.action#sharing) requires authors to make all data underlying the findings described in their manuscript fully available without restriction, with rare exception (please refer to the Data Availability Statement in the manuscript PDF file). The data should be provided as part of the manuscript or its supporting information, or deposited to a public repository. For example, in addition to summary statistics, the data points behind means, medians and variance measures should be available. If there are restrictions on publicly sharing data—e.g. participant privacy or use of data from a third party—those must be specified.

Reviewer #1: No

Reviewer #2: No

**Response: Thank you for your guidance**

The anonymised quantitative data set has been uploaded as the supporting document. However the qualitative recordings and transcripts contains potentially sensitive information about the health care workers and their respective hospitals, this will be against the respective hospital ethical review boards and the Makerere University IRB. Attached is the contact for Makerere University IRB in case of further requests for qualitative data requests; [rresearch9@gmail.com](mailto:rresearch9@gmail.com) OR [research@chs.mak.ac.ug](mailto:research@chs.mak.ac.ug)

**Comment:** Could be interesting in any future work to conduct qualitative interviews with patients- specifically those who decline to start ART at the time of hospitalization. Data from the HCW interviews provide some insights into those who opt to start vs. those who do not. Interviews with patients would provide additional interesting insights.

**Response: We thank you for this comment, we also acknowledge this as a limitation and surely patient interviews would provide additional insight.**

**Comment:** The authors may consider providing details of what the current clinical recommendations are for those who are TB/HIV co-infected. In many clinical settings, there is confusion on this point with HCWs unclear on what the guidelines are or discomfort with shifting guidelines. In interviews with the HCWs- was it clear that they were providing clinical care that was in line with the current national or global guidelines?

**Response: Thank you for this suggestion**

**The current WHO guidelines for initiation of ART among patients with HIV suspected to have TB should initiate ART as soon as possible. In those with confirmed TB, ART should be initiated as soon as possible within 2 weeks of starting Anti-TB drugs. Exception is made in patients suspected to have TB or cryptococcal meningitis; ART should be delayed at least until 4 weeks after initiating treatment. (Discussion, Page 16)**

**Comment:** There are clear clinical contra-indications to immediate ART initiation in this group e.g. CCM, TB, critically ill, died as in your Table 3. This group should be removed from your analysis of 'factors associated with early ART'. i.e. The demographics, distance from home etc are only relevant to analysing the patient factors for deferring ART. Otherwise could be confounders.

**Response: Thank you so much for this observation, we have removed TB, CCM from the multivariate analysis of 'factors associated with early ART'. Since Table 3 is rather a descriptive table, showing various reasons for deferring ART we kept them there.**

Comment: You don't discuss all the factors found significant on your Table 4, and I suspect many were chance findings as there isn't a plausible link that comes to mind. Eg, marital status?

**Response Thank you so much for this important suggestion, We have only discussed plausible factors like distance from health facility and education status, Discussion, page 16 and 17**

Comment: Please recheck Education figures, and no CI for employment?

**Response: Thank you for this observation**

**There is one patient who did not have an outcome (ART status at discharge), we put a foot note on table 4 (*) clarifying on the n= 110 instead of 111**

**These figures have now been corrected, thank you so much for the observation**

**The employment variable has no CI because it didn’t go to the multivariate model, we only provided CI to those that made it to the multivariate model**

**Comment:** In your discussion you have to highlight the fact that this was a pragmatic, observational study with the MAJOR confounder between the 2 groups being that those with TB, CCM and early death weren't initiated (appropriately). You shouldn't be tempted to draw any conclusions about lower mortality in a highly selected less sick group initiated early. It would require a carefully designed RCT to adequately answer this Q.

**Response: We acknowledge the short falls of the study design and we have stated that study was not powered to draw conclusions on the difference in the mortality in the limitation section, on page 17**

**Comment:** Table 2> what relevance are WBC, PLT, ALT, AST? Suggest omit

**Response: Thanks for the comment; these have been omitted as suggested**

**Comment:** It would be useful to compare the primary reasons for admission/ final diagnosis between the Early ART & deferred groups if possible to help the reader understand this patient group.

**Response: Data regarding reason of admission/diagnosis rather than TB and CCM, would be very heterogenic and hard to generalize. Since this was an observational study, many patients had more than one reason of admission collected in terms of presenting symptoms, but also multiple final diagnoses which most of them were clinically decided. This information would be very hard to generalize and this is another limitation of the study.**

**Comment:** "The proportion (95% confidence interval) of hospitalized patients with a new HIV diagnosis who initiated ART during hospitalization was calculated as the number of patients initiated on ART out of the total number of hospitalized patients with a new HIV diagnosis admitted in the hospital during the study period." - Surely it's rather the total number recruited for the study?

**Response: Thanks for the comment, we have made the changes as suggested on page 5**

**Comment:** Your qualitative aspect does raise some important programmatic issues.

**Response: Thank you so much , it a our honor**

**Comment:** Only half having CD4 is a limitation

**Thank you for this comment, this is acknowledged and reported as a limitation on page 17**

**Thank you so much**
